# A one-dimensional iodido-bridged Pt^II^/Pt^IV^ mixed-valence complex cation with a hydrogen sulfate counter-anion

**DOI:** 10.1107/S2056989018016158

**Published:** 2018-11-22

**Authors:** Nobuyuki Matsushita

**Affiliations:** a Department of Chemistry & Research Center for Smart Molecules, Rikkyo University, Nishi-Ikebukuro 3-34-1, Toshima-ku, 171-8501 Tokyo, Japan

**Keywords:** crystal structure, platinum complex, one-dimensional chain complex, iodido-bridged complex, Pt(II,IV) mixed-valence, *MX*-chain

## Abstract

The title compound, *catena*-poly[[[bis­(ethyl­enedi­amine-κ^2^ *N*,*N*′)platinum(II)]-μ-iodido-[bis­(ethyl­enedi­amine-κ^2^ *N*,*N*′)platinum(IV)]-μ-iodido] tetra­(hydrogen sulfate) dihydrate], {[Pt^II^(C_2_H_8_N_2_)_2_][Pt^IV^I_2_(C_2_H_8_N_2_)_2_](HSO_4_)_4_·2H_2_O}_*n*_, has a linear chain structure comprising alternating platinum cations with mixed-valent oxidation states of +II/IV. Square-planar [Pt(en)_2_]^2+^ cations and elongated octa­hedral *trans*-[PtI_2_(en)_2_]^2+^ cations (en is ethyl­enedi­amine) are stacked alternately parallel to the *b* axis, and are bridged by the I ligands. The Pt site of the [Pt^II/IV^(en)_2_] units is located on a twofold rotation axis. The I site, which is located on the same twofold rotation axis, is equally disordered over two positions. The Pt and I sites form a straight ⋯I—Pt^IV^—I⋯Pt^II^⋯ chain, with Pt^IV^—I bond lengths of 2.7202 (6) and 2.6917 (6) Å, and Pt^II^⋯I contacts of 3.2249 (6) and 3.2534 (6) Å. The mixed-valence state of the Pt site is expressed by the structural parameter *δ* = (Pt^IV^–I)/(Pt^II^⋯I), with values of 0.843 and 0.827 for the two independent I atoms. In the crystal structure, the cationic columnar structure is stabilized by hydrogen bonds of the type N—H⋯O between the amine groups of the Pt complex chains and the disordered hydrogen sulfate counter anions, and between the amine groups and water mol­ecules of crystallization. In addition, O—H⋯O hydrogen bonds between the hydrogen sulfate anions and water mol­ecules of crystallization and between the hydrogen sulfate anions themselves consolidate the crystal packing.

## Abstract 2

Straight ⋯I—Pt^IV^—I⋯Pt^II^⋯ chains are observed in the mixed-valent title salt. Extensive hydrogen bonding involving the amino groups, hydrogen sulfate counter-anions and water mol­ecules of crystallization stabilizes the crystal packing.

## Chemical context   

The title mixed-valence compound, [Pt^II^(en)_2_][Pt^IV^I_2_(en)_2_](HSO_4_)_4_·2H_2_O (en is ethyl­enedi­amine, C_2_N_2_H_8_), (I), is a member of the family of one-dimensional halogenido-bridged mixed-valence metal complexes, formulated as [*M* ^II^(*AA*)_2_][*M* ^IV^ *X*_2_(*AA*)_2_]*Y*_4_ [*M* ^II^/*M* ^IV^ = Pt^II^/Pt^IV^; Pd^II^/Pd^IV^; Ni^II^/Ni^IV^; Pd^II^/Pt^IV^; Ni^II^/Pt^IV^; *X* = Cl, Br, I; *AA* = NH_2_(CH_2_)_2_NH_2_, *etc*.; *Y* = ClO_4_^−^, BF_4_^−^, *X* ^−^, *etc*.], which are often referred to as *MX-chains* and are typical mixed-valence compounds belonging to class II in the classification of Robin & Day (1967 ▸). *MX-chains* have attracted much inter­est because of their one-dimensional mixed-valence electron systems, as described in a previous report (Matsushita, 2006 ▸).

The metal–halogen distances in crystals of *MX-chains* characterize their physical properties based on the mixed-valence electronic state. The X-ray structure determination of (I) was performed to gain structural information for *MX-chains* and to compare (I) with chlorido- and bromido-bridged Pt^II^/Pt^IV^ mixed-valence complexes with a hydrogen sulfate counter-anion, *i.e*. [Pt^II^(en)_2_][Pt^IV^ *X*_2_(en)_2_](HSO_4_)_4_ (*X* = Cl, Br) (Matsushita *et al.*, 1992 ▸; Matsushita, 2003 ▸).
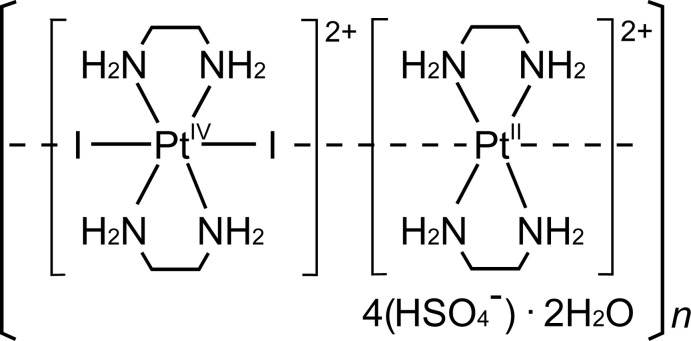


## Structural commentary   

**Figure 1:**
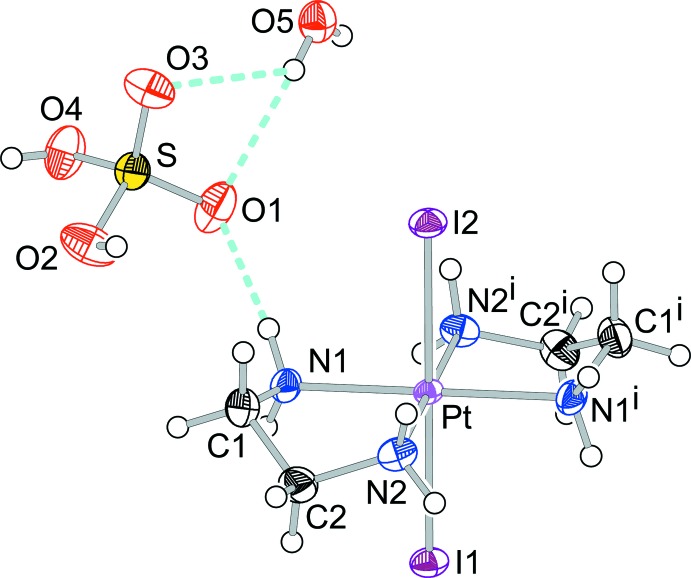
The structures of the mol­ecular components of (I), showing the atomic numbering scheme. Displacement ellipsoids are drawn at the 50% probability level for non-H atoms. Light-blue dashed lines represent N—H⋯O and O—H⋯O hydrogen bonds. Each site of atoms I1 and I2 is half occupied. [Symmetry code: (i) 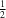 − *x*, *y*, 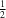 − *z*].

**Figure 2:**
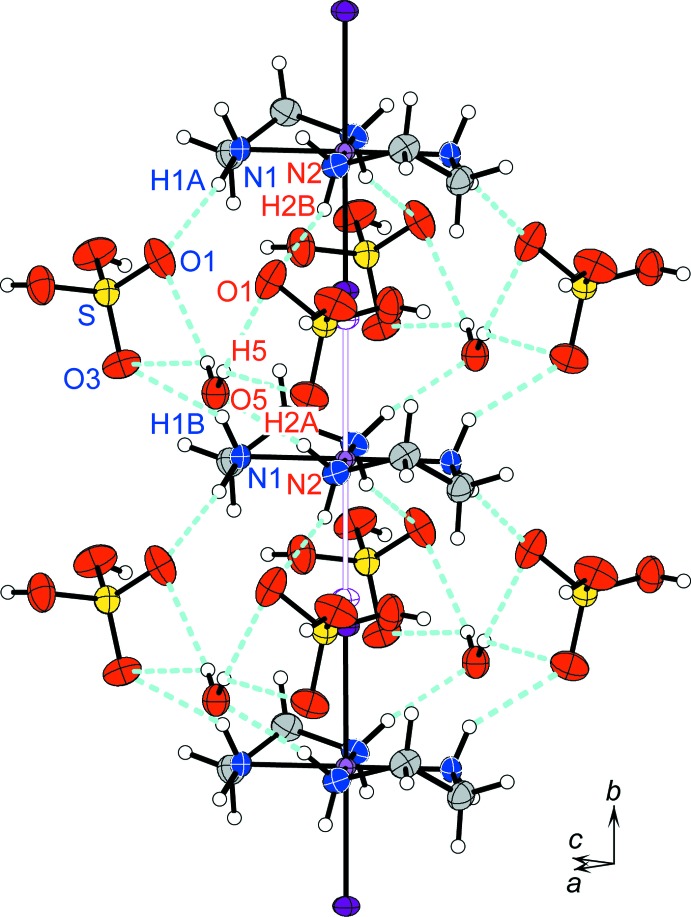
A view of the columnar structure of compound (I), running parallel to the *b* axis. Displacement ellipsoids are drawn at the 50% probability level for non-H atoms. The violet hollow ellipsoids of I atoms and the violet hollow lines between Pt and I atoms represent the disordered part of the ⋯I—Pt^IV^—I⋯Pt^II^⋯ chain. Light-blue dashed lines represent hydrogen bonds.

The structures of the mol­ecular components of (I) are displayed in Fig. 1 ▸. The asymmetric unit of (I) comprises half of a Pt-complex moiety, [Pt^II^(en)_2_]^2+^ or [Pt^IV^I_2_(en)_2_]^2+^, one HSO_4_^−^ anion, and a half-mol­ecule of water. The Pt and I atoms of the Pt-complex moiety and the O atom of the water mol­ecule are located on twofold rotation axes. The hydrogen sulfate anion lies on a general position. As shown in Fig. 2 ▸, the structure of (I) is built up of columns extending parallel to the *b* axis, composed of square-planar [Pt(en)_2_]^2+^ cations and elongated octa­hedral *trans*-[PtI_2_(en)_2_]^2+^ cations stacked alternately and bridged by the I ligands. The Pt and I atoms form an infinite straight ⋯I—Pt^IV^—I⋯Pt^II^⋯ chain. The same straight chains are also observed in [Pt^II^(en)_2_][Pt^IV^ *X*_2_(en)_2_](HSO_4_)_4_ (*X* = Cl, Br) (Matsushita *et al.*, 1992 ▸; Matsushita, 2003 ▸). The title salt (I) is, however, not isotypic with these hydrogen sulfates of the chlorido- and bromido-bridged complexes whereas the latter structures show isotypism with each other.

The I sites in (I) are not located at the exact midpoint between adjacent Pt sites and thus are equally disordered over two sites close to the midpoint. Consequently, the Pt site is occupationally disordered over the Pt^II^ and Pt^IV^ atoms. The valence ordering of the Pt site in (I) belongs to one of three different classes of the order–disorder problem pointed out by Keller (1982 ▸). The structure of (I) can be regarded as being of a one-dimensionally ordered structure type, with the other two directions being in a disordered state. The structural order–disorder situation of the Pt site in (I) has also been observed in the structures of a number of other *MX-chains* (Endres *et al.*, 1980 ▸; Beauchamp *et al.*, 1982 ▸; Cannas *et al.*, 1983 ▸; Yamashita *et al.*, 1985 ▸; Matsushita *et al.*, 1992 ▸, 2017 ▸; Toriumi *et al.*, 1993 ▸; Huckett *et al.*, 1993 ▸; Matsushita, 2003 ▸, 2005*a* ▸,*b* ▸, 2015 ▸; Matsushita & Taira, 2015 ▸).

With respect to the two sites for the disordered I atoms, the shorter Pt—I distances are assigned to Pt^IV^—I and the longer ones to Pt^II^⋯I contacts, as follows: I—Pt^IV^—I; Pt—I1 = 2.7202 (6) Å, Pt—I2 = 2.6917 (6) Å; I⋯Pt^II^⋯I; Pt⋯I1 = 3.2249 (6) Å, Pt⋯I2 = 3.2534 (6) Å. Other bond lengths and angles are collated in Table 1 ▸.

The structural parameters indicating the mixed-valence state of the Pt site, expressed by δ = (Pt^IV^–I)/(Pt^II^⋯I), are 0.843 and 0.827 for I1 and I2, respectively. These values are smaller than those of [Pt(pn)_2_][PtI_2_(pn)_2_](ClO_4_)_4_ (pn is 1,2-di­amino­propane) (0.937; Breer *et al.*, 1978 ▸), [Pt(pn)_2_][PtI_2_(pn)_2_]I_4_ (0.940; Endres *et al.*, 1980 ▸), [Pt(tn)_2_][PtI_2_(tn)_2_](ClO_4_)_4_ (tn is 1,3-di­amino­propane) (0.95; Cannas *et al.*, 1984 ▸), [Pt(en)_2_][PtI_2_(en)_2_](ClO_4_)_4_ (0.919; Endres *et al.*, 1979 ▸), but are comparable with those of [Pt(NH_3_)_4_][PtI_2_(NH_3_)_4_](HSO_4_)_4_·2H_2_O (0.834; Tanaka *et al.*, 1986 ▸), [Pt(en)_2_][PtI_2_(en)_2_](C_8_H_17_SO_3_)_4_·2H_2_O (0.839 and 0.858; Matsushita, 2015 ▸), and somewhat larger than those of [Pt(en)_2_][PtI_2_(en)_2_](HPO_4_)(H_2_PO_4_)I·3H_2_O (0.812 and 0.818; Matsushita, 2006 ▸).

## Supra­molecular features   

Hydrogen bonds in (I) (Table 2 ▸) stabilize the columnar structure composed only of cationic complexes, as shown in Fig. 2 ▸. A [Pt^II/IV^(en)_2_] unit is bound to an adjacent Pt-complex unit in the column by four hydrogen-bond linkages as follows: two linkages N1—H1*A*⋯O1—S—O3⋯H1*B*—N1 and two linkages N2—H2*A*⋯O5—H5⋯O1⋯H2*B*—N2. In addition, the donor group O5—H5 is hydrogen-bonded to atom O3, and forms a three-centre hydrogen-bond. Such hydrogen-bonded linkages are common structural motifs of *MX-chains* (Matsushita, 2003 ▸, 2005*a* ▸,*b* ▸, 2006 ▸, 2015 ▸; Matsushita *et al.*, 1992 ▸, 2017 ▸; Matsushita & Taira, 2015 ▸).

**Figure 3:**
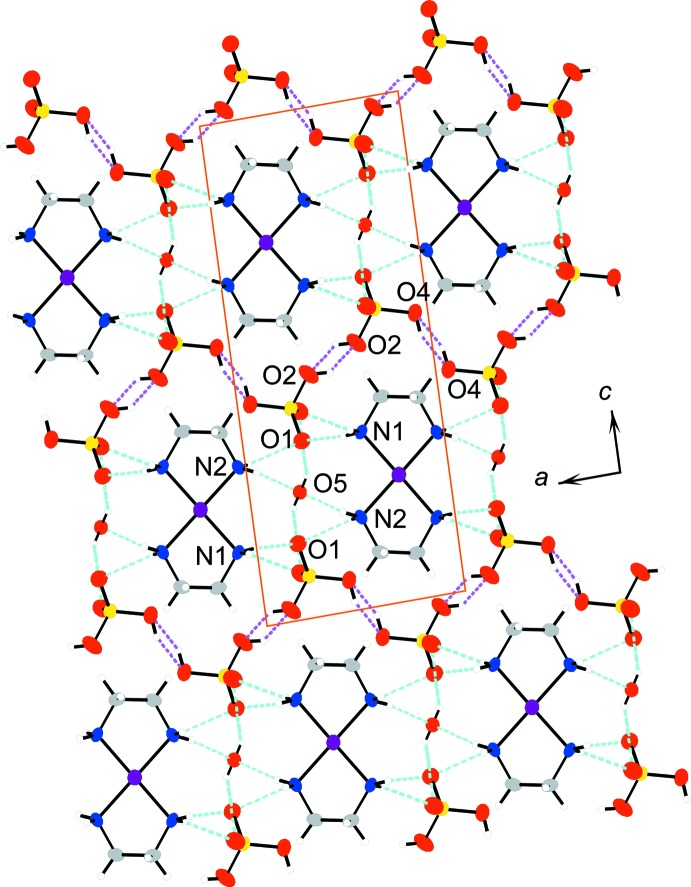
The crystal packing of compound (I), projected on the *ac* plane. Magenta dashed lines represent hydrogen bonds between the hydrogen sulfate ions. Light-blue dashed lines represent the other hydrogen bonds. Solid orange lines indicate the unit cell.

As a result of the inter­columnar hydrogen-bond linkages, N1—H1*A*⋯O1⋯H2*B*—N2 between the Pt-complex columns and hydrogen sulfate ions, and N2—H2*A*⋯O5⋯H2*A*—N2 between the Pt-complex columns and the water mol­ecule of crystallization, represented by light-blue dashed lines in Fig. 3 ▸, the columns are organized in layers parallel to the *ab* plane.

The layers are connected along the direction of the *c* axis by two very short hydrogen bonds (Table 2 ▸) between hydrogen sulfate ions as follows: O2—H2⋯O2^vi^ and O4—H4⋯O4^vii^, represented by magenta dashed lines in Fig. 3 ▸. Atom pairs O2 and O2^vi^ and O4 and O4^vii^ both are related by inversion centers. Thus, atoms H2 and H4 are equally disordered over two sites between atoms O2 and between atoms O4, respect­ively. One-dimensional hydrogen-bonded chains of hydrogen sulfate anions run along the *a*-axis direction. Similar hydrogen-bonded chains of hydrogen sulfate anions are observed in [Pt^II^(en)_2_][Pt^IV^ *X*_2_(en)_2_](HSO_4_)_4_ (*X* = Cl, Br) (Matsushita *et al.*, 1992 ▸; Matsushita, 2003 ▸). In the hydrogen sulfate ion, the lengths of the S—O(H) bonds [S—O2 = 1.499 (2) Å, S—O4 = 1.491 (2) Å] are longer than those of the S—O bonds [S—O1 = 1.448 (2) Å, S—O3 = 1.432 (2) Å]. This difference in the S—O bond lengths supports the fact that both O2 and O4 are bonded to a hydrogen atom, however in a disordered manner. A similar difference in the lengths of the S—O and S—O(H) bonds is also observed in [Pt^II^(en)_2_][Pt^IV^ *X*_2_(en)_2_](HSO_4_)_4_ (*X* = Cl, Br) (Matsushita *et al.*, 1992 ▸; Matsushita, 2003 ▸). In these hydrogen sulfates, however, the hydrogen atoms of the hydrogen sulfate anions, which also hydrogen-bond to neighbouring hydrogen sulfate anions, are not disordered. The lengths of the S—O(H) bond and the S—O bond for the acceptor O atom are 1.494 (10) and 1.420 (8) Å, respectively, for the chlorido-bridged complex and 1.45 (2) and 1.35 (3) Å for the bromido-bridged complex. These longer and shorter lengths for the S—O bonds indicate that the hydrogen atoms of the hydrogen sulfate ions are not disordered.

The intra­columnar, inter­columnar and inter­layer hydrogen-bonds, as discussed above, stabilize the crystal packing in (I).

## Synthesis and crystallization   

A preparation procedure for the title salt was previously reported (Matsushita *et al.*, 1989 ▸). In the literature, the obtained salt was originally reported as a tetra­hydrate. The present X-ray crystallographic study, however, reveals the salt to be a dihydrate. Probably, the amount of water mol­ecules of the salt was overestimated at that time due to the hygroscopic nature of the polycrystalline sample because the salt was obtained from a concentrated sulfuric acid solution. The powder X-ray diffraction pattern simulated on the basis of the present single-crystal data is in good agreement with the experimental data reported previously for the powder sample.

## Refinement   

Crystal data, data collection and structure refinement details are summarized in Table 3 ▸. Atoms I1, I2 and H2 and H4 are each disordered over two positions and were modelled with an occupancy factor of 0.5. Hydrogen atoms were placed in geometrically calculated positions and refined as riding, with C—H = 0.97 Å, N—H = 0.89 Å, and O—H = 0.82 Å, and with *U*_iso_(H) = 1.2*U*_eq_(C,N) and 1.5*U*_eq_(O). Hydrogen atoms bonded to O atoms were calculated by the HFIX 147 command of *SHELXL* (Sheldrick, 2015*b* ▸). Evaluation of the S—O2 bond length for atom H2, the S—O4 bond length for atom H4, and the O3⋯O5 and O1⋯O5 hydrogen bonds together with other hydrogen-bonding inter­actions showed the expected behaviour, and therefore the localization of these H atoms was considered to be correct. The maximum and minimum electron density peaks are located 0.67 and 0.17 Å, respectively, from atom Pt.

## Supplementary Material

Crystal structure: contains datablock(s) global, I. DOI: 10.1107/S2056989018016158/wm5469sup1.cif

Structure factors: contains datablock(s) I. DOI: 10.1107/S2056989018016158/wm5469Isup2.hkl

CCDC reference: 1878955

Additional supporting information:  crystallographic information; 3D view; checkCIF report

## Figures and Tables

**Table 1:** Selected geometric parameters (Å, °)

Pt—N2	2.055 (2)	N2—C2	1.492 (4)
Pt—N1	2.057 (2)	C1—C2	1.501 (4)
Pt—I2	2.6917 (6)	S—O3	1.432 (2)
Pt—I1	2.7202 (6)	S—O1	1.448 (2)
Pt—I1^i^	3.2249 (6)	S—O4	1.491 (2)
Pt—I2^ii^	3.2534 (6)	S—O2	1.499 (2)
N1—C1	1.499 (4)		
			
N2—Pt—N1	83.23 (10)	N2—C2—C1	107.3 (2)
N2—Pt—I2	90.27 (6)	O3—S—O1	113.41 (15)
N1—Pt—I2	89.96 (6)	O3—S—O4	111.27 (15)
N2—Pt—I1	89.73 (6)	O1—S—O4	105.28 (14)
N1—Pt—I1	90.04 (6)	O3—S—O2	109.72 (14)
C1—N1—Pt	108.82 (17)	O1—S—O2	110.33 (16)
C2—N2—Pt	108.60 (18)	O4—S—O2	106.55 (15)
N1—C1—C2	107.7 (2)		

Symmetry codes: (i) 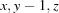; (ii) 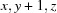.

**Table 2:** Hydrogen-bond geometry (Å, °)

*D*—H⋯*A*	*D*—H	H⋯*A*	*D*⋯*A*	*D*—H⋯*A*
N1—H1*A*⋯O1	0.89	2.01	2.895 (3)	173
N1—H1*B*⋯O3^ii^	0.89	2.29	3.057 (3)	145
N2—H2*A*⋯O5^iii^	0.89	2.03	2.905 (3)	169
N2—H2*B*⋯O1^iv^	0.89	2.39	3.132 (3)	141
O5—H5⋯O1^v^	0.82	2.28	3.032 (4)	152
O5—H5⋯O3^v^	0.82	2.36	2.936 (3)	128
O2—H2⋯O2^vi^	0.82	1.92	2.595 (5)	139
O4—H4⋯O4^vii^	0.82	1.83	2.560 (5)	148

Symmetry codes: (ii) 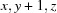; (iii) 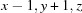; (iv) 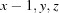; (v) 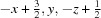; (vi) 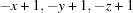; (vii) 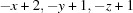.

**Table 3:** Experimental details

Crystal data	
Chemical formula	[Pt(C_2_H_8_N_2_)_2_][PtI_2_(C_2_H_8_N_2_)_2_](HSO_4_)_4_·2H_2_O
*M*_r_	1308.70
Crystal system, space group	Monoclinic, *P*2/*n*
Temperature (K)	296
*a*, *b*, *c* (Å)	7.2964 (2), 5.9451 (2), 18.2253 (7)
β (°)	92.318 (1)
*V* (Å^3^)	789.93 (5)
*Z*	1
Radiation type	Mo *K*α
μ (mm^−1^)	11.15
Crystal size (mm)	0.50 × 0.40 × 0.35

Data collection	
Diffractometer	Rigaku R-AXIS RAPID imaging plate
Absorption correction	Multi-scan (*ABSCOR*; Rigaku, 1995 ▸)
*T*_min_, *T*_max_	0.010, 0.020
No. of measured, independent and observed [*I* > 2σ(*I*)] reflections	16218, 2733, 2541
*R*_int_	0.048
(sin θ/λ)_max_ (Å^−1^)	0.746

Refinement	
*R*[*F* ^2^ > 2σ(*F* ^2^)], *wR*(*F* ^2^), *S*	0.024, 0.046, 1.21
No. of reflections	2733
No. of parameters	106
H-atom treatment	H-atom parameters constrained
Δρ_max_, Δρ_min_ (e Å^−3^)	2.12, −1.69

Computer programs: *RAPID-AUTO* (Rigaku, 2015 ▸), *SHELXT* (Sheldrick, 2015*a* ▸), *SHELXL2014*/7 (Sheldrick, 2015*b* ▸) and *DIAMOND* (Brandenburg, 2018 ▸).

## supplementary crystallographic information

### Crystal data

**Table:**

[Pt(C_2_H_8_N_2_)_2_][PtI_2_(C_2_H_8_N_2_)_2_](HSO_4_)_4_·2H_2_O	*F*(000) = 614
*M**_r_* = 1308.70	*D*_x_ = 2.751 Mg m^−^^3^
Monoclinic, *P*2/*n*	Mo *K*α radiation, λ = 0.71075 Å
*a* = 7.2964 (2) Å	Cell parameters from 14539 reflections
*b* = 5.9451 (2) Å	θ = 3.1–32.1°
*c* = 18.2253 (7) Å	µ = 11.15 mm^−^^1^
β = 92.318 (1)°	*T* = 296 K
*V* = 789.93 (5) Å^3^	Block, gold
*Z* = 1	0.50 × 0.40 × 0.35 mm

### Data collection

**Table:**

Rigaku R-AXIS RAPID imaging plate diffractometer	2733 independent reflections
Radiation source: X-ray sealed tube	2541 reflections with *I* > 2σ(*I*)
Graphite monochromator	*R*_int_ = 0.048
Detector resolution: 10.00 pixels mm^-1^	θ_max_ = 32.0°, θ_min_ = 3.1°
ω scans	*h* = −10→10
Absorption correction: multi-scan (ABSCOR; Rigaku, 1995)	*k* = −8→8
*T*_min_ = 0.010, *T*_max_ = 0.020	*l* = −27→27
16218 measured reflections	

### Refinement

**Table:**

Refinement on *F*^2^	Secondary atom site location: difference Fourier map
Least-squares matrix: full	Hydrogen site location: inferred from neighbouring sites
*R*[*F*^2^ > 2σ(*F*^2^)] = 0.024	H-atom parameters constrained
*wR*(*F*^2^) = 0.046	*w* = 1/[σ^2^(*F*_o_^2^) + 0.4688*P*] where *P* = (*F*_o_^2^ + 2*F*_c_^2^)/3
*S* = 1.21	(Δ/σ)_max_ < 0.001
2733 reflections	Δρ_max_ = 2.12 e Å^−^^3^
106 parameters	Δρ_min_ = −1.68 e Å^−^^3^
0 restraints	Extinction correction: SHELXL-2014/7 (Sheldrick 2015b), Fc^*^=kFc[1+0.001xFc^2^λ^3^/sin(2θ)]^-1/4^
Primary atom site location: dual	Extinction coefficient: 0.0149 (4)

### Special details

**Table:**

Geometry. All esds (except the esd in the dihedral angle between two l.s. planes) are estimated using the full covariance matrix. The cell esds are taken into account individually in the estimation of esds in distances, angles and torsion angles; correlations between esds in cell parameters are only used when they are defined by crystal symmetry. An approximate (isotropic) treatment of cell esds is used for estimating esds involving l.s. planes.

### Fractional atomic coordinates and isotropic or equivalent isotropic displacement parameters (Å^2^)

**Table:**

	*x*	*y*	*z*	*U*_iso_*/*U*_eq_	Occ. (<1)
Pt	0.2500	0.98097 (2)	0.2500	0.01577 (6)	
I1	0.2500	1.43852 (10)	0.2500	0.02522 (15)	0.5
I2	0.2500	0.52821 (9)	0.2500	0.02578 (15)	0.5
N1	0.4066 (3)	0.9807 (3)	0.34641 (14)	0.0247 (5)	
H1A	0.4946	0.8775	0.3443	0.030*	
H1B	0.4593	1.1145	0.3533	0.030*	
N2	0.0356 (3)	0.9826 (3)	0.31975 (14)	0.0254 (5)	
H2A	−0.0501	1.0791	0.3036	0.030*	
H2B	−0.0144	0.8463	0.3215	0.030*	
C1	0.2853 (4)	0.9293 (5)	0.40872 (16)	0.0317 (6)	
H1C	0.3428	0.9795	0.4548	0.038*	
H1D	0.2643	0.7685	0.4118	0.038*	
C2	0.1070 (4)	1.0503 (5)	0.39440 (17)	0.0313 (6)	
H2C	0.0201	1.0094	0.4310	0.038*	
H2D	0.1260	1.2117	0.3965	0.038*	
S	0.73971 (11)	0.53489 (12)	0.41680 (4)	0.02863 (16)	
O1	0.7066 (3)	0.6617 (4)	0.34976 (13)	0.0463 (6)	
O2	0.6291 (3)	0.6293 (4)	0.47713 (14)	0.0474 (6)	
H2	0.5242	0.5798	0.4734	0.071*	0.5
O3	0.7028 (4)	0.2994 (4)	0.40865 (14)	0.0431 (6)	
O4	0.9361 (3)	0.5761 (4)	0.43844 (13)	0.0422 (5)	
H4	0.9504	0.5661	0.4832	0.063*	0.5
O5	0.7500	0.2587 (5)	0.2500	0.0321 (7)	
H5	0.7954	0.3426	0.2200	0.048*	

### Atomic displacement parameters (Å^2^)

**Table:**

	*U*^11^	*U*^22^	*U*^33^	*U*^12^	*U*^13^	*U*^23^
Pt	0.01352 (8)	0.01268 (8)	0.02104 (8)	0.000	−0.00028 (5)	0.000
I1	0.0248 (3)	0.0168 (3)	0.0340 (3)	0.000	0.0010 (2)	0.000
I2	0.0292 (3)	0.0151 (3)	0.0328 (3)	0.000	−0.0006 (2)	0.000
N1	0.0218 (12)	0.0239 (11)	0.0277 (12)	0.0006 (8)	−0.0072 (10)	−0.0011 (8)
N2	0.0208 (12)	0.0247 (11)	0.0309 (13)	0.0010 (8)	0.0061 (10)	0.0030 (9)
C1	0.0394 (18)	0.0325 (15)	0.0231 (14)	0.0034 (13)	−0.0013 (13)	0.0019 (11)
C2	0.0362 (18)	0.0317 (14)	0.0264 (15)	0.0053 (12)	0.0074 (13)	−0.0005 (11)
S	0.0268 (4)	0.0302 (4)	0.0288 (4)	0.0001 (3)	−0.0014 (3)	−0.0002 (3)
O1	0.0403 (14)	0.0602 (15)	0.0382 (14)	0.0105 (12)	−0.0009 (11)	0.0158 (12)
O2	0.0473 (15)	0.0433 (14)	0.0533 (16)	−0.0060 (11)	0.0242 (12)	−0.0156 (11)
O3	0.0501 (15)	0.0326 (12)	0.0471 (16)	−0.0032 (10)	0.0096 (12)	−0.0097 (10)
O4	0.0283 (12)	0.0560 (14)	0.0419 (14)	−0.0073 (10)	−0.0041 (10)	0.0105 (12)
O5	0.0262 (17)	0.0400 (18)	0.0300 (19)	0.000	0.0011 (14)	0.000

### Geometric parameters (Å, º)

**Table:**

Pt—N2	2.055 (2)	N2—H2A	0.8900
Pt—N2^i^	2.055 (2)	N2—H2B	0.8900
Pt—N1^i^	2.057 (2)	C1—C2	1.501 (4)
Pt—N1	2.057 (2)	C1—H1C	0.9700
Pt—I2	2.6917 (6)	C1—H1D	0.9700
Pt—I1	2.7202 (6)	C2—H2C	0.9700
Pt—I1^ii^	3.2249 (6)	C2—H2D	0.9700
Pt—I2^iii^	3.2534 (6)	S—O3	1.432 (2)
I1—I2^iii^	0.5332 (6)	S—O1	1.448 (2)
I1—Pt^iii^	3.2249 (6)	S—O4	1.491 (2)
I2—I1^ii^	0.5332 (6)	S—O2	1.499 (2)
I2—Pt^ii^	3.2534 (6)	O2—H2	0.8200
N1—C1	1.499 (4)	O4—H4	0.8200
N1—H1A	0.8900	O5—N2^iv^	2.905 (3)
N1—H1B	0.8900	O5—H5	0.8200
N2—C2	1.492 (4)		
			
N2—Pt—N2^i^	179.45 (11)	C1—N1—Pt	108.82 (17)
N2—Pt—N1^i^	96.77 (10)	C1—N1—H1A	109.9
N2^i^—Pt—N1^i^	83.23 (10)	Pt—N1—H1A	109.9
N2—Pt—N1	83.23 (10)	C1—N1—H1B	109.9
N2^i^—Pt—N1	96.77 (10)	Pt—N1—H1B	109.9
N1^i^—Pt—N1	179.92 (11)	H1A—N1—H1B	108.3
N2—Pt—I2	90.27 (6)	C2—N2—Pt	108.60 (18)
N2^i^—Pt—I2	90.27 (6)	C2—N2—H2A	110.0
N1^i^—Pt—I2	89.96 (6)	Pt—N2—H2A	110.0
N1—Pt—I2	89.96 (6)	C2—N2—H2B	110.0
N2—Pt—I1	89.73 (6)	Pt—N2—H2B	110.0
N2^i^—Pt—I1	89.73 (6)	H2A—N2—H2B	108.4
N1^i^—Pt—I1	90.04 (6)	N1—C1—C2	107.7 (2)
N1—Pt—I1	90.04 (6)	N1—C1—H1C	110.2
I2—Pt—I1	180.0	C2—C1—H1C	110.2
N2—Pt—I1^ii^	90.27 (6)	N1—C1—H1D	110.2
N2^i^—Pt—I1^ii^	90.27 (6)	C2—C1—H1D	110.2
N1^i^—Pt—I1^ii^	89.96 (6)	H1C—C1—H1D	108.5
N1—Pt—I1^ii^	89.96 (6)	N2—C2—C1	107.3 (2)
I2—Pt—I1^ii^	0.0	N2—C2—H2C	110.3
I1—Pt—I1^ii^	180.0	C1—C2—H2C	110.3
N2—Pt—I2^iii^	89.73 (6)	N2—C2—H2D	110.3
N2^i^—Pt—I2^iii^	89.73 (6)	C1—C2—H2D	110.3
N1^i^—Pt—I2^iii^	90.04 (6)	H2C—C2—H2D	108.5
N1—Pt—I2^iii^	90.04 (6)	O3—S—O1	113.41 (15)
I2—Pt—I2^iii^	180.0	O3—S—O4	111.27 (15)
I1—Pt—I2^iii^	0.0	O1—S—O4	105.28 (14)
I1^ii^—Pt—I2^iii^	180.0	O3—S—O2	109.72 (14)
I2^iii^—I1—Pt	180.0	O1—S—O2	110.33 (16)
I2^iii^—I1—Pt^iii^	0.000 (1)	O4—S—O2	106.55 (15)
Pt—I1—Pt^iii^	180.0	S—O2—H2	109.5
I1^ii^—I2—Pt	180.0	S—O4—H4	109.5
I1^ii^—I2—Pt^ii^	0.0	N2^iv^—O5—H5	109.5
Pt—I2—Pt^ii^	180.0		

Symmetry codes: (i) −*x*+1/2, *y*, −*z*+1/2; (ii) *x*, *y*−1, *z*; (iii) *x*, *y*+1, *z*; (iv) *x*+1, *y*−1, *z*.

### Hydrogen-bond geometry (Å, º)

**Table:**

*D*—H···*A*	*D*—H	H···*A*	*D*···*A*	*D*—H···*A*
N1—H1*A*···O1	0.89	2.01	2.895 (3)	173
N1—H1*B*···O3^iii^	0.89	2.29	3.057 (3)	145
N2—H2*A*···O5^v^	0.89	2.03	2.905 (3)	169
N2—H2*B*···O1^vi^	0.89	2.39	3.132 (3)	141
O5—H5···O1^vii^	0.82	2.28	3.032 (4)	152
O5—H5···O3^vii^	0.82	2.36	2.936 (3)	128
O2—H2···O2^viii^	0.82	1.92	2.595 (5)	139
O4—H4···O4^ix^	0.82	1.83	2.560 (5)	148

Symmetry codes: (iii) *x*, *y*+1, *z*; (v) *x*−1, *y*+1, *z*; (vi) *x*−1, *y*, *z*; (vii) −*x*+3/2, *y*, −*z*+1/2; (viii) −*x*+1, −*y*+1, −*z*+1; (ix) −*x*+2, −*y*+1, −*z*+1.
